# Prevalence of erosive tooth wear and associated risk factors in Colombian adolescents

**DOI:** 10.1590/1807-3107bor-2024.vol38.0050

**Published:** 2024-06-24

**Authors:** Viviana AVILA, Edgar Orlando BETLRÁN, Andrea CORTÉS, Margarita USUGA-VACCA, Jaime Eduardo CASTELLANOS PARRAS, David DIAZ-BAEZ, Stefania MARTIGNON

**Affiliations:** (a) Universidad El Bosque, Unica - Caries Research Unit, Research Department, Universidad El Bosque, Bogotá, Colombia.; (b) Universidad El Bosque, Grupo de Virología, Vicerrectoría de Investigaciones, Bogotá, Colombia.; (c) Universidad El Bosque, School of Dentistry, Unit of Basic Oral Investigation, Bogotá, Colombia.

**Keywords:** Tooth Erosion, Tooth Wear, Adolescent, Prevalence, Risk Factors

## Abstract

Accurate determination of the prevalence of erosive tooth wear (ETW) and associated risk factors in adolescents can inform clinical management guidelines. The aim of this analytical cross-sectional study was to estimate the prevalence, severity, and risk factors of ETW in adolescents aged 12–15 years in the municipality of Usaquén in Bogotá, Colombia. Two calibrated examiners clinically assessed ETW using Basic Erosive Wear Examination (BEWE) Index (range: 0–3). All tooth surfaces (excluding proximal) were scored to allow estimation of the Highest (0–3) and Total BEWE (sum of Highest BEWE score per sextant: 0–18) scores per patient. Sociodemographic characteristics, ETW risk factors, and caries severity (ICDAS-epi-merged) were evaluated, and their association with the presence of ETW (indicated by a Highest BEWE score of 2–3) was examined using preliminary analyses and logistic regression models. The study sample included 454 adolescents (mean age: 13.5±1.1 years; female: 61.7%), and the prevalence of ETW was 71.6%. The majority of participants exhibited a Highest BEWE score of 3 (58.0%) and a Total BEWE score ≤8 (84.3%). The preliminary analysis showed an association between the presence of ETW and age, caries, and brushing teeth before eating (p-value < 0.05). Risk factors for ETW included always brushing teeth before eating [adjusted Prevalence Ratio (PRa) 1.31, p-value=0.014], presence of extensive carious lesions (PRa 1.23, p-value = 0.024), male gender (PRa 1.14, p-value = 0.028), and age > 14 years (PRa 1.17, p-value = 0.009). Although ETW was highly prevalent, most Colombian adolescents exhibited low Total BEWE scores. ETW was associated with frequent fruit intake, age, toothbrushing habits, caries lesions, and sex.

## Introduction

Erosive tooth wear (ETW), defined as the cumulative loss of mineralized tooth substance, is primarily caused by the chemical process of dental erosion upon exposure of the tooth surface to acids not derived from oral bacteria. Moreover, the physical/mechanical processes of attrition and abrasion have also been shown to play a role in the development and rapid progression of the condition.^1,2^ A diagnosis of ETW can be made following thorough oral examination and assessment of all risk factors, and the condition can be classified thereafter using the Basic Erosive Wear Examination Index (BEWE).^1,3^

The prevalence of ETW increases with age, particularly if managed inadequately, and previous studies have reported prevalence rates of 20%–40% in the permanent dentition.^4^ Adolescents also represent a key population of interest, with several studies reporting high prevalence rates in this age-group despite the relatively shorter duration of exposure of the permanent dentition to the oral environment.^4,5^ In Colombia, the prevalence of ETW was found to be approximately 57.3% (using the O’Sullivan index) in children aged 10–15 years^7^ and 73.0% (using the BEWE index) in individuals aged 18–25 years.^8^

The presence of ETW and its predisposing factors can compromise tooth function and aesthetics.^4,5^ Previous studies have demonstrated an association between ETW and sociodemographic status, general/oral health status, dietary habits, and oral health care habits.^2,8^ Therefore, the current study aimed to evaluate the prevalence, severity, and risk factors of ETW among adolescents from schools in the municipality of Usaquén in Bogotá, Colombia.

## Methodology

This observational analytical cross-sectional study, approved by the Institutional Ethics Committee of the Universidad El Bosque (Nr. 014-2018), included school-children aged between 12–15 years and studying in Usaquén, Bogotá between August 2018–June 2019. Usaquén, where the university campus is located, is one of largest (5^th^ in terms of area and 6^th^ in terms of population) out of 20 municipalities in Bogotá.^15^ It is one of the 30 most populated cities globally, with over seven million inhabitants, and ranks 1^st^ in terms of transportation challenges faced.^16^

Calculation of the sample size using the proportion formula and assuming an ETW prevalence rate of 50%, a type-I error of 0.05, and an estimated statistical power of 80% (OpenEpi software) yielded a study sample of 454 adolescents.^4,17^

**Figure 1:**
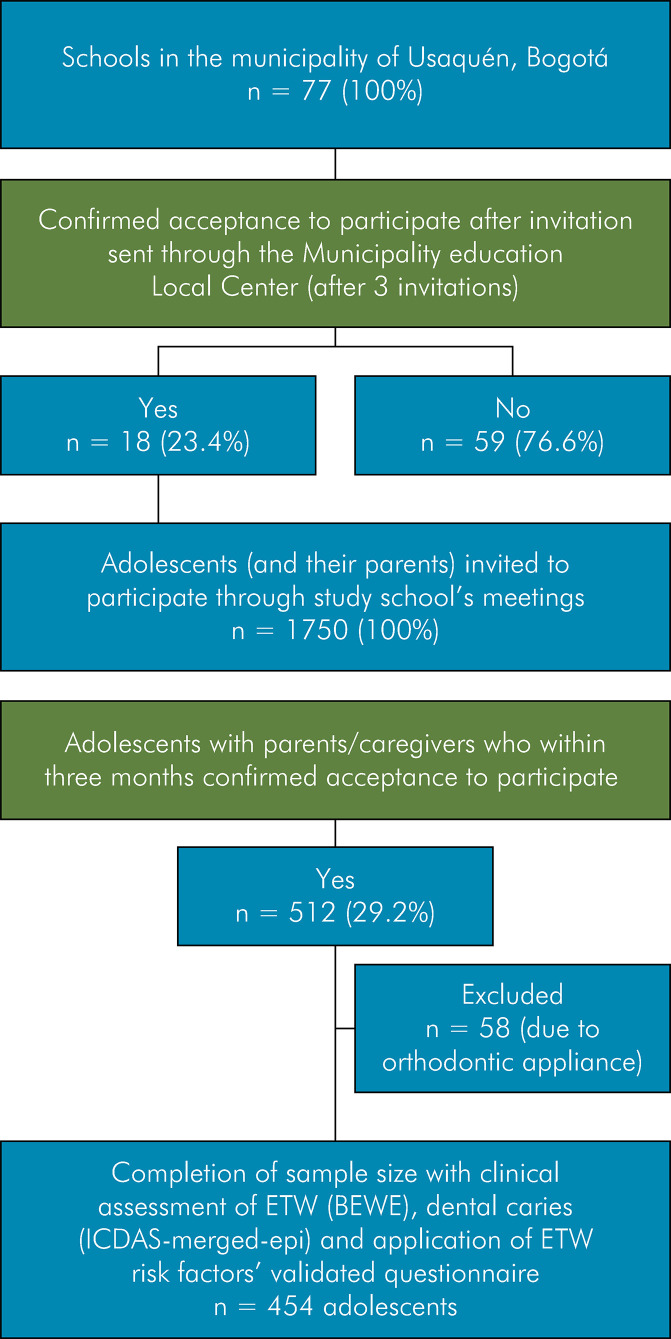
Study flowchart showing sampling procedure.

Data on the number of adolescent schools in Usaquén and their demographic characteristics [e.g., number of children per school, school-funding type (*i.e.*, state or private)] were collected from the Local Education Administrative Centre (LEAC). All schools in Usaquén (n = 77) were invited to take part in the study via the LEAC, and a meeting explaining the study and inviting adolescents and their parents to participate was conducted in all schools that agreed to partake. Individuals who were willing to participate and signed the consent (parents) and assent (adolescents) forms within one month of invitation were recruited using a non-probabilistic sampling method until the sample size was achieved. Patients exhibiting systemic diseases, advanced periodontal disease, tumors in the oral cavity, and those using orthodontic appliances were excluded from the study. Figure 1 shows the study flowchart.

Sociodemographic characteristics such as age, sex, and family socioeconomic status [evaluated using the dwelling-socioeconomic status classification (*i.e.*, low; medium; high) published by the National Administrative Department of Statistics]^18^ were recorded, and the participant’s height and weight were measured to allow calculation of body mass index (BMI). The participants were then interviewed and the protective/risk factors of ETW were recorded using a validated questionnaire,^14^containing 24 items including general/oral health (5 items), dietary habits and frequency of intake of fruit with erosive potential (15 items), oral care habits (4 items), and the presence of carious lesions. Clinical examination of the participant’s ETW and dental caries status was carried out during school-hours in the morning using portable dental units (without triple air syringe), headlamps, intraoral mirror, ball-ended probe, tweezer and cotton rolls.

The BEWE Index was used to classify ETW, and all assessments were carried out by two examiners (VA, EB) who attended a three-day training and calibration course (lead by an expert in the BEWE visual criteria), that included theoretical and practical components, counting with a guided assessment, discussion, and re-assessment of ETW on natural teeth and in patients.^3^ The inter/intra-examiner reliability Kappa scores ranged between 0.71 and 0.86. During clinical assessment, the buccal, occlusal/incisal, and lingual/buccal surfaces of the teeth were examined and scored using the BEWE index (0 = tooth surfaces without wear; 1 = initial loss of surface texture; 2 = surfaces with a distinct defect and loss of hard tissue < 50% of the surface area, and 3 = surfaces with loss of hard tissue >50% of the surface area).^3^

Two other examiners calibrated in the ICDAS visual caries criteria (AC, MU; inter-/intra-examiner reliability Kappa values ≥0.7) used the ICDAS-epi-merged visual criteria (no air drying; ICCMS™) to evaluate the presence and severity of carious lesions [sound surfaces: no caries; initial/non-cavitated caries (ICDAS 1-2: D_I_); moderate caries (microcavity/dentinal shadow; ICDAS 3-4: D_M_); and extensive/cavitated caries (ICDAS 5-6:D_E_)] on the tooth surfaces.

### Data analysis

The Highest and Total BEWE values were calculated to allow assessment of the severity and distribution of ETW in the study sample.^3^ The former corresponded to the maximum BEWE score (range: 0 to 3)^3^, with participants being categorized into ETW absent/minimal (0–1) or present (2–3), while the latter corresponded to the sum of the Highest BEWE scores per sextant in the oral cavity (range: 0 to 18).^8,3^

Age was dichotomized into early (12–13 years) and intermediate (14–15 years) adolescence, as per the American Academy of Paediatrics and the Colombian Ministry of Health criteria.^19^ Body mass index (BMI) was classified into percentiles, as follows: underweight (percentile 10); underweight risk (percentiles > 10–25); healthy weight for age (percentiles > 25–75); overweight (percentiles > 75–90); and obese (percentile > 90).

Descriptive statistics including the prevalence of ETW and the distribution of the Highest BEWE score by sextant, teeth, and tooth surfaces were reported. Frequencies and percentages were calculated for all qualitative variables.

Multivariate analyses were used to identify risk factors after grouping the participants into two categories based on the absence (Highest BEWE score 0-1) or presence (Highest BEWE score 2–3) of ETW. A preliminary analysis of the association between the dependent variable (ETW absence/presence) and socio-demographic characteristics, clinical features, general/oral health status, and dietary and oral care habits was carried out using the Chi square test or the exact Fisher’s test to enable evaluation of likelihood trends and frequency distributions. The direction and magnitude of the associations were evaluated using Poisson regression models with scale parameters adjusted using the Chi squared models to allow estimation of the adjusted prevalence ratios (PRa) and 95% confidence intervals (CI). The final multivariate models were created by retaining all relevant clinical and biological variables and gradually eliminating variables identified in the univariate analysis using the stepwise regression technique with an entry and exit probability of 0.1 and 0.25, respectively. A p-value < 0.05 was considered statistically significant, and all statistical analyses were carried out using STATA 12.

The differences between individuals who agreed to participate and those who did not were assessed by comparing the demographic characteristics of private vs. state funded schools (Chi square test; p-value < 0.05).

## Results

The study sample included 454 adolescents with a mean age of 13.5 ± 1.1 years. The majority of participants were girls (n = 280; 61.7%); from middle social-economic strata (66.7%); had BMI values appropriate for their age (77.3%); and attended private schools (68.7%). Analysis comparing participating vs. non-participating children with respect to municipality’s type of school (private/state) disclosed a significant difference, as 89.8% of children in non-participating schools attended private schools (p < 0.05).

**Figure 2:**
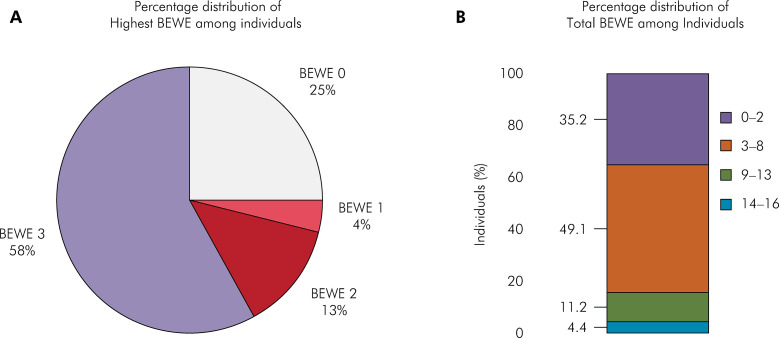
Distribution (in percentage) of (A) Highest BEWE and (B) Total BEWE scores among participants.

The prevalence of ETW was approximately 71.6%, and the most frequently observed Highest BEWE score was 3 (58.1%). Moreover, ETW most frequently affected the anterior upper and lower sextants of the oral cavity, and the majority of the study sample exhibited Total BEWE scores ranging between 3–8 (49.1%) followed by 0–2 (35.2%), suggesting localized severe dentition wear. Figure 2 shows the distribution of the Highest and Total BEWE scores as well as the presence of tooth wear by sextant.

**Figure 3:**
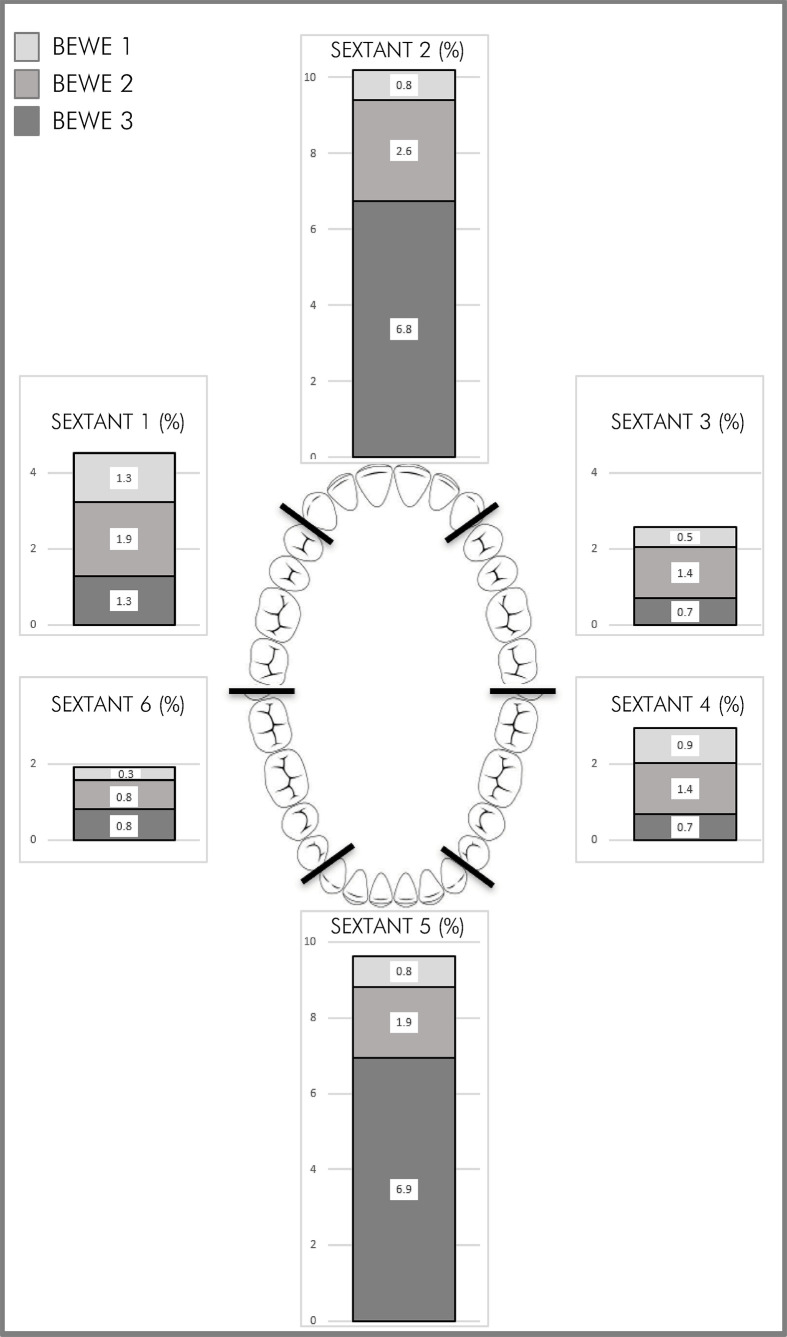
Distribution (in percentage) of the presence of ETW by Highest BEWE score in the dental arch and sextant.

Examination of the distribution of ETW by tooth surface showed a symmetric tooth wear pattern on the right and left sides (shown in Figure 3). The buccal tooth surfaces were most frequently affected by ETW (74.9%), with the central incisors being the most commonly affected teeth (lower left: 8.1%; lower right: 7.51%; upper right: 6.4%, and upper left: 6.3%; shown in Figure 3).

The majority of participants exhibited at least one initial caries lesion (81.5%), followed by moderate (31.1%) and extensive caries lesions (9.9%). Table 2 summarizes the questionnaire responses. Among the general health questions, 9.6% of the participants reported heartburn, 3.8% reported frequent vomiting, 8.9% reported regurgitation, and 8.6% presented gastric symptoms. Among the dental health questions, 14.6% of the participants exhibited tooth bruxism or clenching and 22.4% reported presence of dry mouth. In response to the questions on dietary habits and the consumption of fruits with erosive potential, 28.8% of participants reported consuming lime daily, 64.1% reported consuming at least two portions of whole fruit or natural fruit juice daily, and 86.3% took more than 10 minutes to eat/drink. Additionally, 46.9% of the participants consumed chewing gum and acidic gummies at least twice a day, while 65.6% reported consuming dairy products. The assessment of oral care habits showed that 87.4% of participants did not brush their teeth before eating, 85.2% brushed their teeth at least twice a day, 74.6% brushed their teeth at night before going to bed, and 71.1% had visited their dentist within the last year.

**Table 2:** Preliminary analysis of the association between presence of ETW† and the participants’ general and oral health status, diet, and oral care habits.

Variable	Categories	Absence of ETW		ETW		p-value

		n	%	n	%	
General and oral health status						
Presence of heartburn	No	121	93.8	314	96.6	0.176
	Yes	8	6.2	11	3.4	
Presence of vomiting	No	127	98.4	318	97.8	0.677
	Yes	2	1.6	7	2.2	
Presence of regurgitation or return of food/liquids to the mouth	No	125	96.9	306	94.2	0.229
	Yes	4	3.1	19	5.8	
Presence of bruxism, grinding, or clenching of teeth	No	122	94.6	295	90.8	0.181
	Yes	7	5.4	30	9.2	
Dry mouth or decreased salivary flow in the mouth	No	112	86.8	295	90.8	0.213
	Yes	17	13.2	30	9.2	
Dietary habits						
Frequency of consumption of whole fruit/glasses of natural fruit juice per day	≤1 time per day	42	32.6	111	34.2	0.147
	2 times per day	71	55.0	192	59.1	
	≥3 times per day	16	12.4	22	6.8	
Total time (in minutes) spent per day eating fruits/drinking natural juices	1–10 minutes	52	40.3	158	48.6	0.259
	11–30 minutes	67	51.9	148	45.5	
	>30 minutes	10	7.8	19	5.8	
Frequency of chewing sour jellybeans/gum or sour candy	≤1 time per day	74	57.4	167	51.4	0.243
	2times per day	38	29.5	94	28.9	
	≥3 times per day	17	13.2	64	19.7	
Frequency of consumption of milk, yoghurt, cheese, or other dairy products	≤times per day	47	36.4	109	33.5	0.161
	2times per day	50	38.8	153	47.1	
	≥3 times per day	32	24.8	63	19.4	
Frequency of consumption of acidic products such as lime/lemon with meals.	≤1 time per day	116	89.9	282	86.8	0.367
	2times per day	9	7.0	22	6.8	
	≥3 times per day	4	3.1	21	6.5	
Frequency of consumption of fruits with erosive potential						
Strawberry	≤1 time per day	114	88.4	297	91.4	0.599
	2 times per day	8	6.2	14	4.3	
	≥3 times per day	7	5.4	14	4.3	
Lime	≤1 time per day	109	84.5	282	86.8	0.149
	2 times per day	15	11.6	22	6.8	
	≥3 times per day	5	3.9	21	6.5	
Lulo fruit	≤1 time per day	118	91.5	302	92.9	0.591
	2 times per day	5	3.9	14	4.3	
	≥3 times per day	6	4.7	9	2.8	
Tangerine	≤1 time per day	110	85.3	293	90.2	0.298
	2 times per day	13	10.1	20	6.2	
	≥3 times per day	6	4.7	12	3.7	
Green mango	≤1 time per day	122	94.6	303	93.2	0.141
	2 times per day	3	2.3	18	5.5	
	≥3 times per day	4	3.1	4	1.2	
Green apple	≤1 time per day	116	89.9	301	92.6	0.590
	2 times per day	9	7.0	18	5.5	
	≥3 times per day	4	3.1	6	1.8	
Passion fruit	≤1 time per day	124	96.1	319	98.2	0.418
	2 times per day	3	2.3	3	0.9	
	≥3 times per day	2	1.6	3	0.9	
Blackberry	≤1 time per day	123	95.3	309	95.1	0.977
	2 times per day	4	3.1	10	3.1	
	≥3 times per day	2	1.6	6	1.8	
Orange	≤1 time per day	114	88.4	298	91.7	0.384
	2 times per day	12	9.3	24	7.4	
	≥3 times per day	3	2.3	3	0.9	
Pineapple	≤1 time per day	121	93.8	316	97.2	0.118
	2 times per day	7	5.4	6	1.8	
	≥3 times per day	1	0.8	3	0.9	
Oral care habits						
Time elapsed since last dental appointment	≤1 year	99	76.7	224	68.9	0.097
	>1 year	30	23.3	101	31.1	
Toothbrushing frequency	<2 times per day	20	15.5	47	14.5	0.244
	=2 times per day	75	58.1	166	51.1	
	>2 times per day	34	26.4	112	34.5	
Toothbrushing before going to sleep	No	36	29.5	77	23.7	0.203
	Yes	91	70.5	248	76.3	
Toothbrushing before eating	Never or rarely	121	93.8	276	84.9	0.028*
	Frequently	5	3.9	22	6.8	
	Always	3	2.3	27	8.3	

ETW: erosive tooth wear; p-value < 0.05: statistically significant; Chi square or Fisher’s exact test were used for analysis.

Tables 1 and 2 show the results of the preliminary analysis. Age (p-value = 0.013), the presence of severe carious lesions (p-values = 0.018), and brushing teeth before eating (p-value = 0.028) were significantly associated with the presence of ETW (p-value < 0.05; shown in Table 1), although no such associations were observed with other general/oral health characteristics and dietary habits (shown in Table 2).

**Table 1:** Preliminary analysis of the association between the presence of ETW† and the participants’ socio-demographic & clinical (caries) characteristics.

Variable	Absence of ETW		Presence of ETW		p-value

	n	%	n	%	
Sex					
Male	41	31.8	133	40.9	0.071
Female	88	68.2	192	59.1	
Age (years)					
12–13	96	74.4	202	62.2	0.013*
14–15	33	25.6	123	37.8	
Type of school					
Private	94	72.9	218	67.1	0.230
State funded	35	27.1	107	32.9	
Body mass index					
Risk of low weight	17	13.2	47	17.5	0.594
Appropriate weight	101	78.3	249	76.6	
Overweight or obese	11	8.6	19	5.8	
Socio-economic strata					
Low	19	14.7	63	19.4	0.325
Middle	83	64.3	220	67.7	
High	9	7.0	9	2.7	
No information	18	14.0	33	10.2	
Presence of ICDAS Initial caries lesions					
No	21	16.3	63	19.4	0.442
Yes	108	83.7	262	80.6	
Presence of ICDAS Moderate caries lesions					
No	93	72.1	220	67.7	0.361
Yes	36	27.9	105	32.3	
Presence of ICDAS Extensive caries lesions					
No	123	95.3	286	88.0	0.018*
Yes	6	4.7	39	12.0	

ETW: erosive tooth wear; ICDAS: International Caries Detection and Assessment System. p-value < 0.05: statistically significant; Chi square test used for analysis.

Table 3 shows the results of the multivariate regression analysis. Consumption of pineapple twice a day [PRa 0.63; 95%CI: 0.46–0.87; p-value = 0.005), age > 14 years (PRa 1.17; 95%CI: 1.04–1.33; p-value = 0.009); brushing teeth before eating (PRa 1.31; 95% CI:1.05–1.63; p-value = 0.014); presence of severe carious lesions (PRa 1.23; 95%CI: 1.02–1.48; p-value = 0.024); and male gender (PRa 1.14; 95%CI: 1.01–1.28; p-value = 0.028) were found to be significantly associated with an increased likelihood of the presence of ETW.

**Table 3:** Evaluation of the direction and magnitude of the association between variables related to the presence of ETW† using a Poisson regression model.

Variable	PRa [95%CI]	p-value
Age (years)		
12–13 years	1	0.009*
14 to 15 years	1.17 [1.04–1.33]	
Toothbrushing before eating		
Never or hardly ever	1	0.168
Frequently	1.18 [0.93–1.49]	
Always	1.31 [1.05–1.63]	0.014*
Presence of ICDAS Extensive carious lesions		
No	1	0.024*
Yes	1.23 [1.02–1.48]	
Sex		
Female	1	0.028*
Male	1.14 [1.01–1.28]	
Time elapsed since last dental appointment (year)		
≤ 1	1	0.877*
> 1	1.01 [0.88–1.15]	

ETW: erosive tooth wear; CDAS: International Caries Detection and Assessment System; PRa: adjusted Prevalence Ratio; CI: Confidence interval; *Statistical significance of adjusted Prevalence Ratio (PRa) and the 95% confidence intervals (CI).

## Discussion

The current study estimated the prevalence, severity, and risk factors of ETW among adolescents in the municipality of Usaquén in Bogotá using a validated questionnaire.^14^ The findings showed that ETW was frequently observed among this group of adolescents, although the distribution of wear within the mouth was localized (as shown by the high frequency of low Total BEWE scores). Furthermore, ETW was associated with frequent fruit intake, age, toothbrushing habits, the presence of carious lesions, and sex.

The relatively low proportion of schools providing consent for participation (23.4%), a municipality’s lower proportion of participating children from private schools when compared to that of non-participating schools, and the non-probabilistic sampling technique used limited the representativeness of the study sample. These may be considered as limitations of the study and can potentially be attributed to the low response rates observed in Latin American countries, factors related to the quality of life, and the age group of the participants (as adolescence can be a stressful stage in terms of defining an individuals’ behavior, personality, and self-image).^20,21^ Despite these limitations, the study provides valuable insight into a relatively under-researched area in this region and country, and accurately identifies ETW diagnostic and risk criteria using calibrated examiners and a validated questionnaire.^14^

Another limitation of the current study was that the sample size calculation was based on prevalence estimates rather than the independent variables which may have resulted in lower statistical power. However, the use of validated questionnaires increased confidence in the assessment of risk factors for ETW, particularly during the initial stages.^14^ Further longitudinal evaluation of risk factors would enable examination of the causality of ETW progression.^22^ The use of questionnaires in clinical practice can help identify individuals at risk of developing ETW, thereby facilitating adoption of timely preventive measures and/or control of disease progression through comprehensive patient-centered care.^23,24^

The prevalence of ETW observed in the current study (i.e., 71.6%) was consistent with that reported by previous studies using the BEWE index in adolescents in the region. For instance, some studies reported prevalence rates of approximately 57% in Brazil,^25^ 63.9% in Mexico,^26^ and 52.9% Uruguay,^27^ while others reported a prevalence of 73% (using BEWE index) among 18–25-year-old university students in Columbia^8^ and 57.3% (using the O’Sullivan index) among adolescents in the city of Pasto in Colombia.^7^ A systematic review and meta-analysis (2015) found that the prevalence of permanent tooth erosion among children and adolescents (evaluated using mixed indices) was approximately 30.4%,^10^ while another study in South Brazil reported a prevalence rate of 15% (using the BEWE index) among adolescents aged 15–19 years.^28^ The wide variation in the prevalence of ETW among adolescents globally can be attributed to variations in the diagnostic methods (i.e., diagnosis based on photographs or dental models instead of clinical examination) and indices^5^ [e.g., Tooth Wear Index; assesses the severity and location of tooth wear the Lussi Index (assesses the location of erosion and dentin exposure); the O’Sullivan tooth erosion index (assesses the severity, extent of dentin exposure, and location of lesions); and the Visual Exam of Dental Erosion Visual index (a modification of the Lussi Index; assesses erosive wear in enamel and dentin separately)] used.^6,29^

Dental organisations such as the European Federation of Conservative Dentistry, the European Organisation for Caries Research (ORCA),^1^ the Cariology Research Group of the IADR (CRG-IADR),^1^ and the Erosive Tooth Wear Foundation (supported by Kings College London, UK) recommend the use of the BEWE Index as the preferred diagnostic criteria in clinical practice, with the aim of improving reporting of this condition, facilitating timely detection and management, promoting adoption of BEWE globally, standardizing ETW diagnosis, and making global epidemiological data more comparable.

In the current study, the majority of participants (58.1%) exhibited severe ETW (*i.e.*, BEWE 3) on at least one tooth surface, although the Highest proportion of individuals exhibited Total BEWE scores ranging between of 3 - 8 (49.1%) followed by 0 - 2 (35.2%). These results indicate localized severe tooth wear in the mouth of the participants.^3^ The majority of previous studies did not evaluate the Total BEWE score, thereby preventing comparison of this parameter. However, the Highest BEWE scores reported to date indicate the presence of mild^26,28^ or moderate ETW,^30^ and this was consistent with the findings of the current study as well as previous evidence based on indices other than the BEWE.^4,7^ The current study also showed that the buccal surfaces of the central incisor teeth were most frequently affected, and this was in agreement with previous studies that recommended use of these surfaces as indicators for ETW.^31^

Patients exhibiting severe carious lesions exhibited a higher risk of presenting tooth wear and the need for operative care, and this was also consistent with some previous evidence.^13 11^ However, much of the evidence on this association remains inconsistent, with some studies also reporting no relationship between the two dental conditions in school-children from different countries.^32^

In the current study, a higher risk of ETW was observed in adolescents over 14 years of age, consistent with previous evidence, and this could be attributed to the natural history of this cumulative and irreversible condition.^5,35^

Previous studies have reported observing a higher risk of ETW in men and have attributed it to the greater prevalence of factors that favor tooth wear (e.g., increased consumption of erosive drinks, greater masticatory strength, harder toothbrushing, and the tendency to use their teeth as tools) among this population.^11,27,28,35,36^ The effect of free testosterone concentrations in the blood on the etiology of tooth wear has also been proposed as a potential explanation for the higher prevalence of ETW among male adolescents.^37^

Toothbrushing before eating was also found to be associated with an increased risk of ETW and previous studies have suggested that this could likely be related to the protective function of the dental biofilm that decreases the loss of ions from mineralized tissues exposed to acidic challenges.^38^

With regard to dietary factors, the current study found that frequent consumption of pineapple (*i.e.*, twice per day) increased the risk of developing ETW, and this could be attributed to the erosive potential and pH (3.6) of the fruit. This was supported by previous evidence that also demonstrated an association between decreased consumption of fruit and a lower risk of developing ETW.^8^ The frequency and timing of intake of acidic foods (acidic fruits) and beverages like fruit juices (pineapple), carbonated drinks, vinegar, and tea have also been shown to be associated with an increased risk of developing ETW, potentially due to the erosive effects of the acid content of these items.^9^

These findings highlight the importance of accurately identifying risk factors for ETW to allow timely detection of this condition in the adolescent population where tooth wear is detectable at an early stage. This, in turn, can also facilitate appropriate implementation of preventive measures that can preserve the dental structure and minimize severe mineral loss.

## Conclusion

The findings of this study showed a high prevalence of ETW in this group of adolescents, with the presence of the condition being associated with frequent fruit intake, age, toothbrushing habits, presence of carious lesions, and sex. These findings emphasize the importance of actively monitoring the adolescent population for the presence of ETW and its risk factors in clinical practice.
